# Effect of voluntary running on adult hippocampal neurogenesis in cholinergic lesioned mice

**DOI:** 10.1186/1471-2202-10-57

**Published:** 2009-06-05

**Authors:** New Fei Ho, Siew Ping Han, Gavin S Dawe

**Affiliations:** 1 Department of Pharmacology, Yong Loo Lin School of Medicine, National University of Singapore, Centre for Life Sciences (CeLS), Level 4, 28 Medical Drive, 117456, Singapore

## Abstract

**Background:**

Cholinergic neuronal dysfunction of the basal forebrain is observed in patients with Alzheimer's disease and dementia, and has been linked to decreased neurogenesis in the hippocampus, a region involved in learning and memory. Running is a robust inducer of adult hippocampal neurogenesis. This study aims to address the effect of running on hippocampal neurogenesis in lesioned mice, where septohippocampal cholinergic neurones have been selectively eliminated in the medial septum and diagonal band of Broca of the basal forebrain by infusion of mu-p75-saporin immunotoxin.

**Results:**

Running increased the number of newborn cells in the dentate gyrus of the hippocampus in cholinergic denervated mice compared to non-lesioned mice 24 hours after injection of bromodeoxyuridine (BrdU). Although similar levels of surviving cells were present in cholinergic depleted animals and their respective controls four weeks after injection of BrdU, the majority of progenitors that proliferate in response to the initial period of running were not able to survive beyond one month without cholinergic input. Despite this, the running-induced increase in the number of surviving neurones was not affected by cholinergic depletion.

**Conclusion:**

The lesion paradigm used here models aspects of the cholinergic deficits associated with Alzheimer's Disease and aging. We showed that running still increased the number of newborn cells in the adult hippocampal dentate gyrus in this model of neurodegenerative disease.

## Background

The principal cholinergic innervation to the hippocampus arises from the basal forebrain, specifically from the medial septum and diagonal band of Broca (MSDB). Progressive loss of basal forebrain cholinergic cells, marked by reduced cholinergic acetyltransferase (ChAT) levels [1,2], acetylcholinesterase activity [2-4] and p75^NTR ^receptor expression [5], occurs in aging, dementia and neurodegenerative diseases such as Alzheimer's disease (AD) [6,7]. According to the "cholinergic hypothesis of AD" posited more than two decades ago, the symptoms of failing cognitive function associated with AD and advanced age are attributed to cholinergic neuronal dysfunction [8,9]. This idea is backed by studies linking the mnemonic functions of the cortex and hippocampus to the cholinergic system [10,11] and the association of cognitive deficits with the severity of the loss of basal forebrain cholinergic neurones [12,13]. More recently, some authors have proposed that the decline in learning and memory is also related to decreased hippocampal neurogenesis associated with the degeneration of cholinergic neurones [14,15].

Neurogenesis in the dentate gyrus of the hippocampus is governed by a multitude of molecular mitogenic signals, transmitters and trophic factors, acting spatially and temporally to modulate distinct steps in the birth and maturation of the new neurones. Besides the pathological loss of cholinergic function, other physiological factors such as stress [16-19], aging [20-22], and drugs of abuse like nicotine [23], alcohol [24] and opiates [25] can reduce adult neurogenesis. Conversely, factors like antidepressants [26,27], exposure to enriched environments [28-30] and hippocampal-dependent learning [31-34] upregulate adult neurogenesis. One of the most striking inducers of neural progenitor cell division in the dentate gyrus is the simple behavioural act of running [35-38].

It is still unclear as to how or why physical activity specifically elicits neurogenic mechanisms in the hippocampus [30]. It is recognized, however, that wheel running evokes a rhythmic firing pattern, theta rhythm, in the hippocampus [39,40]. The synchronous firing of pacemaker cells, comprising cholinergic and GABAergic neurones originating from the MSDB, generate the theta oscillations [41-49]. These septohippocampal projections heavily innervate the dentate gyrus, forming axosomatic contacts with granule cells and axodendritic contacts with hilar cells within the neurogenic locality [50-52]. Increases in the intensity of movement are correlated with increases in frequency of theta [40,53]. Furthermore, running is also associated with acetylcholine release in the hippocampus [54]. Transgenic mice expressing an inactive form of acetylcholinesterase, and hence expected to have elevated acetylcholine levels, showed increased cell proliferation in the subgranular layer of the dentate gyrus [55]. This evidence suggests that the septohippocampal system may be involved in running-mediated neurogenesis.

Our present study aims to investigate the effects of running on hippocampal neurogenesis in cholinergic lesioned mice, which serves as a model for aspects of AD and age-related dementia. To lesion cholinergic projections to the hippocampus, we employed an immunotoxin. Murine-p75-saporin (Mu-p75-SAP) is a conjugate of saporin toxin and a mouse-specific monoclonal antibody directed against the p75 neurotrophin receptor, which is found predominantly on the cholinergic neurones of the basal forebrain. This allows selective elimination, and spares other cholinergic neurones located elsewhere in the brain, even within the adjacent striatum and nucleus accumbens [56-59]. Injection of the immunotoxin results in a substantial reduction in ChAT activity in both the basal forebrain and hippocampal regions, and a concomitant impairment in learning and memory [60,61]. To assess neurogenesis in the dentate gyrus, we performed experiments with the DNA synthesis marker bromodeoxyuridine (BrdU).

## Results

### Cholinergic lesioning in the MSDB is partial but selective

**Figure 1:**
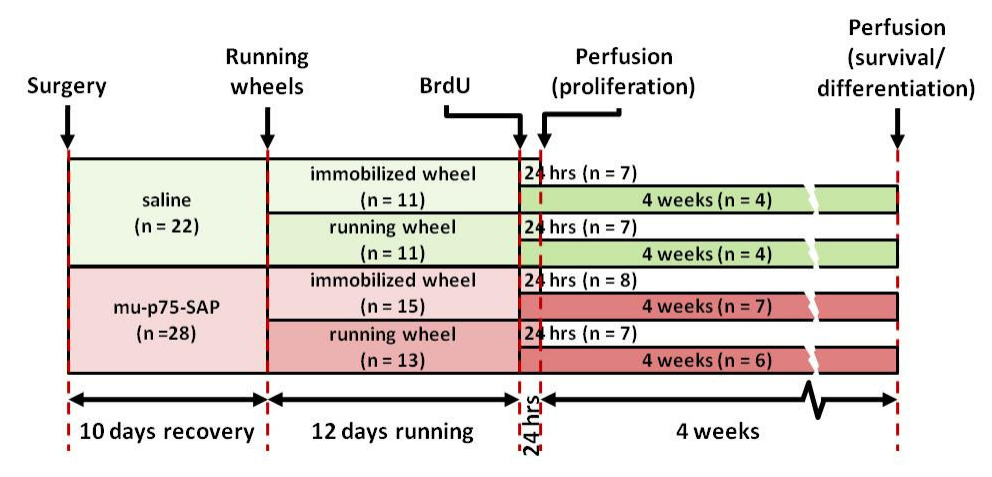
**Schematic representation of the experimental design**. Mice underwent surgery during which they received stereotaxic intracerebroventricular injections of either vehicle or murine-p75-saporin (mu-p75-SAP) immunotoxin. After 10 days for recovery, mice were exposed to either immobilised running wheels or freely moving running wheels. Following 12 days of exposure to the running wheels, the mice received intraperitoneal injections of 5-bromodeoxyuridine (BrdU). The mice were sacrificed for perfusion fixation either 24 hours or 4 weeks after injection of BrdU.

**Figure 2:**
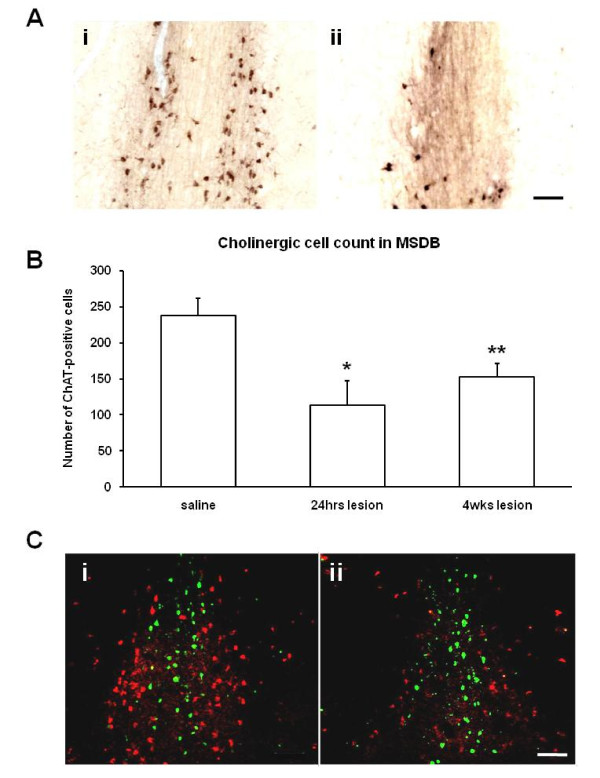
**Murine-p75-saporin injections depleted cholinergic neurones in the MSDB**. (A) Extent of the depletion of cholinergic neurones. Photomicrographs of representative sections through the MSDB immunostained for ChAT visualized with DAB from mice infused with (i) saline and (ii) 3.6 μg/μl of murine-p75-saporin (mu-p75-SAP). Scale bar = 100 μm. (B) Quantification of the extent of the cholinergic depletion. Number of ChAT-immunopositive cells in the MSDB following intracerebroventricular infusion of saline (saline) or mu-p75-SAP (lesion) and sacrifice either 24 hours after the end of running wheel exposure (24 hrs lesion) or 4 weeks after the end of running wheel exposure (4 weeks lesion). Data are mean ± sem; * *p *< 0.05, ** *p *< 0.01. (C) Selectivity of the cholinergic lesions in the MSDB. Confocal micrographs of double immunofluorescence labelling for ChAT (red) and parvalbumin (green), a marker for GABAergic cells, in representative sections from (i) saline control and (ii) mu-p75-SAP infused mice. Parvalbumin-positive cells remained intact despite of loss of ChAT-positive cells. Scale bar = 100 μm.

Adult mice were randomly assigned to running and non-running groups to be sacrificed 24 hours or 4 weeks after injection of BrdU following 12 days of free access to a running wheel or control exposure to an immobilised running wheel (Figure 1). Bilateral stereotaxic infusions of mu-p75-SAP or vehicle were made. After 10 days recovery, running wheels were placed in the cages for 12 days. At a dose of 3.6 μg of immunotoxin, 18 out of 28 lesioned mice survived, a 65% survival rate. This is comparable with a 68% survival previously reported [60]. After 12 days of exposure to the running wheels, mice were injected with BrdU and sacrificed 24 hrs or 4 weeks later (23 days or 50 days after lesioning, respectively). Sections through the MSDB were immunostained for ChAT (Figure 2A) and cholinergic cells were counted. There was no significant difference in the numbers of ChAT-positive neurones between runners and non-runners. We performed one-way ANOVA analysis with Dunnett's post-hoc tests on the number of ChAT labelled cells in the animals that were sham lesioned (238 ± 24) or lesioned and injected with BrdU 24 hrs (114 ± 34) or 4 weeks (153 ± 19) after lesioning. Mu-p75-SAP injections resulted in a significant depletion of cholinergic neurones in the medial septum (F _2,19 _= 5.63, *p *< 0.05) for lesioned groups both 24 hrs and 4 weeks after BrdU injection (*p *< 0.05 and *p *< 0.01, respectively; Figure 2B). GABAergic neurones in the MSDB, identified as parvalbumin-immunopositive cells [62], were not affected by the lesions (Figure 2C). Cholinergic deafferentation did not affect the distance run by the mice. The distance accumulated by each runner daily ranged from 4 km to 25 km. There was no difference in the number of revolutions of the running wheel between the lesioned (272346 ± 3933 revolutions) and the non-lesioned group (246852 ± 2373 revolutions).

### Running increases progenitor cell proliferation in both non-lesioned and lesioned animals

**Figure 3:**
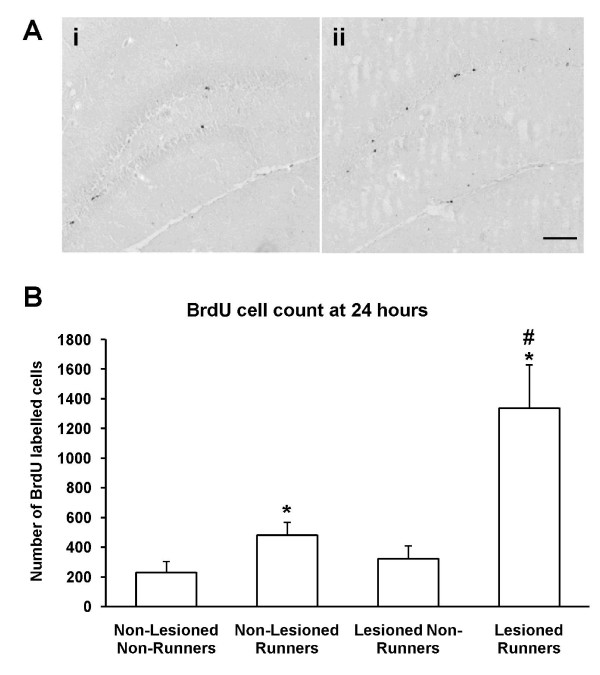
**Running enhances cell proliferation in both non-lesioned and lesioned mice**. (A) Representative photomicrographs of immunohistochemistry for BrdU in the dentate gyrus of (i) a non-runner and (ii) a runner. Scale bar = 200 μm. (B) Running increased the number of BrdU-labelled cells in both the non-lesioned groups and lesioned groups (* *p *< 0.05) 24 hours after BrdU injection. Comparisons between the runners of in the lesioned group and the control group demonstrated that cholinergic denervation significantly potentiated the running-induced increase in BrdU-positive cells (^#^*p *< 0.05). Data are mean ± sem.

Sections through the hippocampus were immunostained for BrdU (Figure 3A). For each brain, we systemically sampled dorsal hippocampal tissue sections from bregma -1.50 mm to -2.50 mm. BrdU-immunopositive cells along the length of the subgranular zone (SGZ) and granule cell layer of the dentate gyrus were counted. Two-way ANOVA showed that both running (F _3,25 _= 15.68, *p *< 0.001) and lesioning (F _3,25 _= 8.88, *p *< 0.01) had significant effects on the number of BrdU-immunopositive cells in the dentate gyrus. There was a significant interaction between running and lesioning (F _3,25 _= 5.69, *p *< 0.05). Post-hoc analysis using independent samples two-tailed t-tests revealed that running increased the number of BrdU-labelled cells in both non-lesioned (*p *< 0.05) and lesioned animals (*p *< 0.05; Figure 3B). Comparisons between the runners in the lesioned group and the control group demonstrated that cholinergic denervation significantly potentiated the running-induced increase in BrdU-immunopositive cells (*p *< 0.05; Figure 3B). There were no significant differences in the length of the dentate gyrus across all treatment groups.

### Running increases survival of progenitor cells despite reduced survival in lesioned animals

**Figure 4:**
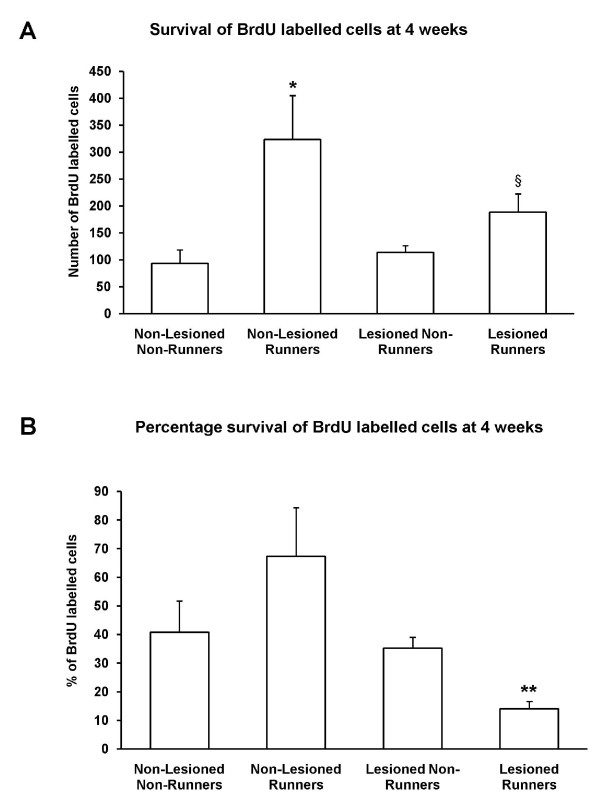
**Both running and lesioning affected survival of newborn cells**. (A) Running increased numbers of BrdU-labelled newborn cells surviving at 4 weeks in both the non-lesioned (* *p *< 0.05) and lesioned groups (§*p *< 0.05). (B) The survival of BrdU-labelled cells at 4 weeks was expressed as a percentage of the number of cells counted at 24 hrs after BrdU injection. The percentage of BrdU-labelled cells that survived for 4 weeks was significantly reduced in the cholinergic lesioned runners (** *p *< 0.001). Data are mean ± sem.

Two-way ANOVA showed that running had a significant effect on the number of BrdU-immunopositive cells surviving at the 4 weeks time point (F _3,17 _= 15.25, *p *< 0.01; Figure 4A). Post-hoc t-tests of unequal variances revealed that this was due to significant increases in BrdU-immunopositive cells surviving in both the non-lesioned (two-tailed: *p *< 0.05) and lesioned runners (one-tailed: *p *< 0.05) (Figure 4A). Two-way ANOVA showed that lesioning had no significant effect on the number of BrdU-immunopositive cells at 4 weeks (F_3,17 _= 2.152, p = 0.161).

To analyse the effect of running and lesioning on the numbers of BrdU-labelled cells over time, we performed a three-way ANOVA. Lesioning (F _7,42 _= 5.91, *p *< 0.05), running (F _7,42 _= 13.851, *p *< 0.001) and time (F _7,42 _= 20.321, *p *< 0.0001) significantly influenced the number of BrdU-labelled cells. There were also significant interactions between lesion and time (F _3,42 _= 5.929, *p *< 0.05) and between running and time (F _3,42 _= 7.536, *p *< 0.01). The three-way interaction between running and lesion over time was significant (F _3,42 _= 4.22, *p *< 0.05), hence we carried out further statistical tests to compare the effect of time within the lesion and running groups. For follow-up analysis, an index of cell survival was calculated by dividing the number of BrdU-labelled cells surviving at the 4 weeks time point by the mean number of labelled cells 24 hours after BrdU administration. Two-way ANOVA showed that lesioning significantly decreased the percentage survival of BrdU-immunopositive cells at the 4 weeks time point (F_3,17 _= 12.84, *p *< 0.01; Figure 4B). There was also a significant interaction between running and lesioning (*p *< 0.05). Post-hoc two-tailed t-tests revealed that cholinergic lesioning significantly decreased the percentage survival of newborn cells in the dentate gyrus of runners compared to non-runners (*p *< 0.001). The proportion of BrdU cells surviving after 4 weeks was marginally, but not significantly, reduced in lesioned runners compared to non-lesioned runners (*p *= 0.053; Figure 4B).

Taken together, these data suggest that without cholinergic inputs, the bulk of progenitors that proliferate in response to running are not able to survive beyond one month.

### Running increases neurogenesis in mice with partial cholinergic denervation

**Figure 5:**
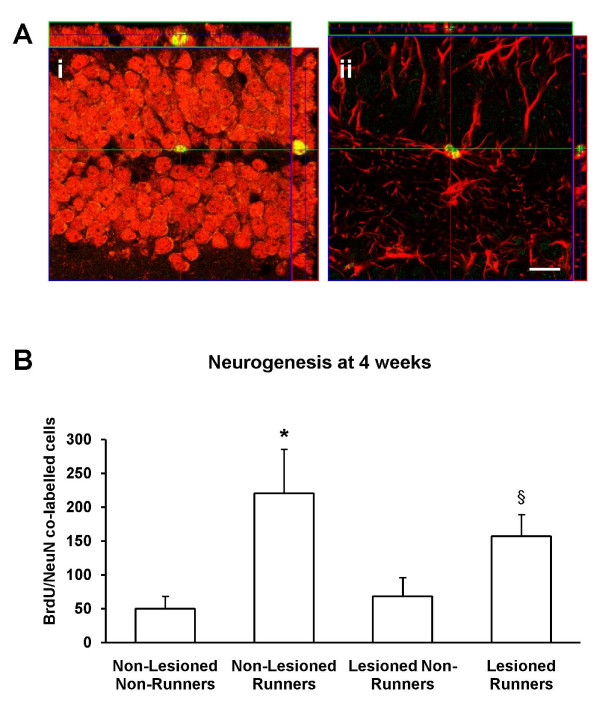
**Running enhances neurogenesis in cholinergic lesioned mice**. (A) Confocal micrographs showing z-series reconstruction of cells double-labelled with (i) BrdU (green) and NeuN (red) and (ii) BrdU (green) and GFAP (red). Scale bar = 20 μm. (B) Sham-lesioned mice allowed access to running wheels exhibit greater numbers of newly generated neurones (* *p *< 0.05, one-tailed) despite discontinuation of running 4 weeks prior to sacrifice. Although not as prominent, the effect of running on neurogenesis was also significant in cholinergic lesioned mice (§*p *< 0.05, one-tailed). Data are mean ± sem.

To determine the phenotype of surviving differentiated newborn cells at the 4 week time point, double immunolabelling was carried out to assay for co-expression of either neuronal specific nucleus protein (NeuN), a marker for mature neurones, or glial acidic fibrillary protein (GFAP), an astroglial marker, with BrdU labelling in cells within the granule cell layer of the DG (Figure 5A). Running had a significant effect on neurogenesis (F _3,17 _= 12.12, *p *< 0.01). In the sham lesioned group, post-hoc t-tests showed that the runners had enhanced neurogenesis (*p *< 0.05). Comparisons between runners and non-runners within the cholinergic deafferented mice showed that, although running was discontinued 4 weeks earlier, the effect of running on neurogenesis was still significant (one-tailed t-test, *p = *0.029) (Figure 5B). Within the runners, lesioning had no effect on neurogenesis (F _3,17 _= 1.126, *p *= 0.286). Neither lesioning (F _3,17 _= 2.676, *p *= 0.120) nor running (F _3,17 _= 2.379, *p *= 0.141) affected the percentage of surviving BrdU-labelled cells that differentiated into neurones.

Astrogenesis remained constant despite the various treatments (lesioning: F _3,17 _= 0.036, *p *= 0.852; running: F _3,17 _= 4.136, *p *= 0.189). Similarly, lesioning (F _3,17 _= 1.433, *p *= 0.248) and running (F _3,17 _= 0.271, *p *= 0.609) did not affect the proportion of astrocytes (Table 1).

**Table 1:** Proliferation, survival and phenotypes of BrdU-positive cells

	Non-lesioned, Non-Runners	Non-lesioned, Runners	Lesioned Non-Runners	Lesioned Runners
Proliferation, 24 hrs	228.5(74.9)	480.0(87.4)	323.5(87.1)	1336.8(293.8)

Survival, 4 wks	93.3(24.9)	323.5(81.4)	113.9(12.3)	188.4(33.9)

Survival (%)	40.8(10.9)	67.4(16.9)	35.2(3.8)	14.1(2.5)

Phenotypes:				

Neurones (%)	57.4(8.7)	67.4(9.3)	68.1(6.8)	80.3(3.9)

Astrocytes (%)	5.6(2.4)	11.0(5.3)	22.6(8.6)	10.2(3.4)

Neurones	50.1(17.9)	220.6(64.5)	68.6(27.5)	157.0(32.0)

Astrocytes	7.6(4.5)	25.1(6.5)	17.1(5.3)	13.6(3.1)

## Discussion

Our findings indicate that running increases adult hippocampal neurogenesis, in spite of cholinergic denervation.

### Lesioning

In contrast to the rat species selective 192-Ig-saporin, which is reported to eliminate virtually all cholinergic cells in the rat forebrain [12,45,63-66], the mouse mu-p75-SAP toxin was not as potent and only resulted in a partial cholinergic lesion. The reduction, nevertheless, was significant and specific, depleting almost half the cholinergic neuronal population but leaving the subpopulation of GABAergic cells intact. The percentage loss in our experiments was comparable to that reported by other groups using mu-p75-SAP in mice [61]. Such partial depletion of the cholinergic neurones in the basal forebrain may more accurately reflect the cholinergic cell loss seen in progressive neurodegenerative diseases, such as Alzheimer's disease, than total cholinergic lesioning.

This selective, partial cholinergic lesioning had no effect on progenitor cell proliferation in mice, as estimated by the BrdU-immunopositive cells present 24 hours after injection of BrdU. This finding is in agreement with a previous study in rats, in which partial lesioning of septohippocampal afferents by intraseptal infusion of N-methyl-D-aspartate (NMDA) did not affect cell proliferation in the hippocampus [67], and is in line with other studies involving pharmacological manipulation of the cholinergic system, which reported no effect on proliferation despite effects on cell survival [68-70]. In contrast, complete transection of the fimbria-fornix, which contains all fibres projecting from the medial septum to the hippocampus in addition to other afferent fibres, was reported to reduce progenitor cell proliferation in the dentate gyrus of rats [71,72]. Moreover, near complete forebrain cholinergic lesioning in rats by injection of high doses of 192-Ig-saporin into the ventricles or both the MSDB and nucleus basalis magnocellularis was also reported to reduce cell proliferation or short-term survival [15]. Together these data suggest that progenitor cell proliferation in the mouse dentate gyrus is relatively insensitive to specific, partial depletion of cholinergic septohippocampal afferents but that complete cholinergic deafferentation can impair adult hippocampal progenitor cell proliferation in rats.

It has been reported that on average, 50% of the newly generated cells in the adult rodent brain die by apoptosis [73,74]. A similar percentage of cell loss occurred in the non-lesioned, non-running group in the present study. However, selective, partial cholinergic lesions did not significantly change the number of surviving cells in the non-running group of mice. This is in contrast to the results of near complete or complete forebrain cholinergic lesions produced by injection of 192-IgG-saporin into the ventricles or both the MSDB and nucleus basalis magnocellularis in rats [14,15]. Likewise, studies conducted using cholinergic agonists and antagonists yielded results which indicated that the neurotransmitter acetylcholine is involved in survival of newborn cells [68-70]. Our present result indicates that survival of the newborn cells of the mouse dentate gyrus is not sensitive to selective, partial lesioning of cholinergic afferents. The greater effect reported in the other studies may be attributable to more complete cholinergic depletion throughout the entire forebrain.

The surviving newborn cells differentiated into cell types expressing either neuroneal or glial cell markers. In our model, the cholinergic system has no effect on the phenotypic fate of progenitor cells in the dentate gyrus, consistent with findings in various previous studies [14,15,67-70].

### Running

In corroboration with findings from previous studies, running increased the number of BrdU-labelled progenitor cells approximately two-fold [30,35,36]. In lesioned animals, running elevated the number of BrdU-positive cells by four-fold. These data suggest that exercise may still increase hippocampal cell proliferation in the face of gradual cholinergic degeneration such as occurs in age-related dementia and progressive neurodegenerative disease such as Alzheimer's disease.

The runners in the sham lesion group also had a significantly greater proportion of surviving BrdU-labelled cells 4 weeks after BrdU injection. About two-thirds of the number of progenitors labelled by BrdU at 24 hours were able to survive for at least one month in the non-lesioned, running group. These data show that running enhanced the survival of newborn cells, similar to findings reported by van Praag and co-workers [36].

Although the running wheel was only present for the first twelve days of the experiment, this initial bout of activity led to an increase in the number of neurones generated in the granule cell layer 4 weeks after removal of the running wheel. This is in agreement with reports that the fate of the newborn cells is decided early [75].

### Interactions between lesioning and running

Interestingly, we observed that cholinergic deafferentation markedly potentiated the running-induced effect on proliferation, leading to an approximately three-fold increase over the non-lesioned runners. Brain-derived neurotrophic factor (BDNF) is reported to be important for regulation of cell proliferation in the adult dentate gyrus [76] and is suggested to be one of the factors involved in mediating the effects of exercise on neurogenesis [77]. Neurotrophins such as NGF, BDNF and NT-3 are synthesized in the hippocampus and undergo retrograde transport to the MSDB where they maintain survival and function of septal neuronal populations [78]. Ablation of basal forebrain cholinergic neurones impairs retrograde transport and may lead to accumulation of neurotrophins in the hippocampus, which could result in an enhanced proliferative response to running.

In contrast to the lesioned non-runners, the lesioned runners group showed a very pronounced reduction in the proportion of newborn cells surviving at 4 weeks. This indicates that selective, partial lesioning of cholinergic afferents, while not sufficient to affect baseline survival of newborn cells in the adult mouse dentate gyrus, can markedly reduce the percentage survival of running-induced newborn cells. In many models of pathology, the robust induction of adult neurogenesis appears to be transient and non-specific. Cell proliferation was increased in animal models of brain insult such as epileptic seizures and stroke but only a small fraction of the newborn cells survived longer than one month [79,80]. Moreover, neuroinflammatory responses, such as occur in response to immunotoxic cholinergic lesions [61], are suggested to be detrimental to the survival of newborn cells [81]. For these reasons, the interaction between running and inflammation caused by the lesions could have potentiated a brief spurt in proliferation, but the viability of the progeny of these precursor cells could not be sustained with the lack of cholinergic input.

Others have reported that physical activity stimulates neurogenic potential by targeting precursor cell division [82,83]. Furthermore, prolonged running can boost the survival of the progeny of these progenitors [82]. Our study was designed to investigate the effects of running on cell proliferation and so running was not continued throughout the four weeks following BrdU injection in our experiments. It would be interesting to observe the effects on survival of BrdU labelled cells in cholinergic ablated mice had running been continued.

Despite the cholinergic depletions, the effect of running-induced elevation in newly generated neurones remained unaltered. In other words, the initial short period of running sufficed to expand the pool of progenitor cells and increase the number of neurones in partial cholinergic denervated mice.

In summary, our results demonstrate that an intact cholinergic system is not an absolute requirement for the maintenance of progenitor cell generation and determination of their lineage. Depleting septohippocampal cholinergic projections may not thwart the pro-proliferative actions of running but acetycholine-regulated signalling may be important in prolonging the viability of the newborn neurones. Given that cholinergic lesions in adult mice lead to impaired learning and memory [60], we used this experimental paradigm to model neurodegenerative diseases that involve loss of cognition such as AD and dementia. Our findings bode well in that physical activity may encourage neurogenesis despite central nervous system cholinergic cellular deficits. That the majority of running-induced progenitors do not survive the onslaught of death-engendering signals such as the absence of cholinergic inputs, shows that additional survival-promoting signals are required to maintain the pool of running-induced newborn cells. Translated loosely, this may be in the form of cognitive challenges, as exemplified in animal models where learning and environmental enrichment promote survival of cells [28,84].

## Conclusion

This study demonstrates that the proliferative effects of running are not affected by reduction in cholinergic input. In the presence of cholinergic depletion, running still resulted in the formation of more neurones despite a reduction in survival of newborn cells.

## Methods

### Animal treatments

Adult female Swiss Albino mice (8–10 weeks) were obtained from the Centre for Animal Resources (CARE), Singapore. The mice were housed in the Animal Holding Unit, National University of Singapore, under a 12 hr light: 12 hr dark cycle, with *ad libitum *access to food and water. The mice were group housed and allowed to acclimatize to their environment for one week prior to commencement of the experiments. All animal procedures were conducted with approval from the Institutional Animal Care and Use Committee (IACUC), National University of Singapore, and were conducted in accordance with the "Guide for the Care and Use of Laboratory Animals" and the "Guidelines for the Care and Use of Mammals in Neuroscience and Behavioral Research", National Research Council, USA.

The mice were anaesthetized with a cocktail of hypnorm and midazolam before undergoing bilateral intracerebroventricular microinjections of murine p75-saporin (mu p75-SAP) (Advanced Targeting Systems, San Diego, CA). Holes were drilled at the following stereotaxic coordinates: AP -1.6 mm, ML ± 1.0 mm, and DV -2.4 mm.

The dose of the toxin was titrated to determine the dose producing the most effective depletion of the cholinergic cells in the MSDB without compromising the well-being of the mice. A dose of 3.6 μg/μl was selected and injected into each ventricle over the course of 5 min using a 1 μl Hamilton syringe with a 26-gauge stainless steel needle (SGE Analytical Science, Austin, TX). The syringe was retracted for 0.1 mm before leaving for an additional 5 min in the ventricle. The mice were allowed 10 days to recover, during which they were weighed daily and given glucose saline infusions. Mice exhibiting severe weight loss (< 80% of their original weight) were euthanized by anaesthetic overdose.

The surviving mice were then randomly assigned to the various treatment groups. They were individually housed in cages equipped with a running wheel each. The control group was exposed to immobilized running wheels to control for the possibility of the running wheels serving as sources of environmental enrichment. The mice were left with their running wheels for 12 days. A photo-sensor was used to monitor the distance run by each mouse.

At the end of 12 days of exercise, BrdU (Sigma, St Louis, MO) at a dose of 20 mg/ml dissolved in saline with 0.06 N NaOH and titrated to a pH of 7.4, was injected intraperitoneally at a concentration of 300 mg/kg, a single high but non-toxic dose [85].

### Immunohistochemistry

The animals were anaesthetized with an overdose of pentobarbital (Nembutal, Ovation Pharmaceuticals, Deerfield, IL) either at (i) 24 hours after BrdU administration to assess for neural cell proliferation or (ii) 4 weeks later for cell survival and differentiation. The mice were then transcardially perfused with 4% paraformaldehyde in 0.1 M of phosphate buffer (pH 7.4), after which the brains were extracted and postfixed overnight in the fixative. The basal forebrains of the mice were then sectioned using a vibratome (Vibroslice, World Precision Instruments, Sarasota, FL) at a thickness of 40 μm prior to immunohistochemical assays. For detailed investigation of cell proliferation, the hippocampi of the mice were processed (LeicaTP1020, Leica Microsystems, Wetzlar, Germany), embedded in paraffin, and cut in 6 μm coronal sections on a rotary microtome (Leitz 1512, Leica Microsystems) before mounting onto slides. For investigation of neural differentiation, the hippocampi of the 4 weeks group were sectioned at a thickness of 40 μm using the vibratome and stored in phosphate buffered saline (PBS) at 4°C until use.

For the paraffin sections, the sections were first de-paraffinized with xylene and subsequently rehydrated with descending concentrations of ethanol prior to incubation in 0.3% hydrogen peroxidase to quench endogenous peroxidase activity. PBS was used for all washing. Sections were then pretreated with 4 N HCL (30 min) and trypsin (1 mg/ml in PBS, 10 min, 37°C) for antigen retrieval. Blocking was carried out using 5% horse serum for 20 min, followed by 30 min of incubation with a mouse monoclonal anti-BrdU antibody (1:200, Neomarkers, Fremont, CA). Sections were then incubated with biotinylated secondary horse anti-mouse antibody for 30 min, and avidin-biotin complex for another 30 min according to the manufacturer's instructions (ABC system, Vector Laboratories, Burlingame, CA), with nickel intensified diaminobenzadine as a chromogen (Vector Laboratories). The slides were rinsed in tap water, dehydrated with 95% and 100% ethanol before washing with xylene, and mounted.

For the vibratome sections, immunofluoroscence double-labelling was carried out on the free- floating sections. The sections were pretreated with 2 N HCl before blocking in 5% goat serum. The primary antibodies used were rat monoclonal anti-BrdU (1:200, Accurate Chemical, Westbury, NY), mouse monoclonal anti-NeuN (1:200, Chemicon, Temucula, CA) and rabbit polyclonal anti-GFAP (1:400, DakoCytomation, Glostrup, Denmark). The secondary antibodies used were Cy2 goat anti-rat (1: 200, Jackson Immunoresearch West Grove, PA), Alexa-Fluor 594 goat anti-mouse and goat anti-rabbit (1:200, Molecular Probes, Eugene, OR). The sections were mounted with Pro-Long anti-fade reagent (Molecular Probes) before being coverslipped.

To label cholinergic neurones in the basal forebrain sections, goat polyclonal anti-ChAT antibody (Chemicon) was used with biotinylated donkey-anti-goat secondary antibody (1:200, goat ABC staining system, Santa Cruz Biotechnology, Santa Cruz, CA) and nickel-enhanced DAB as chromogen. Random but corresponding samples were taken from the medial septum sections of each of the non-lesioned and lesioned groups to carry out double-immunofluorescence labelling of ChAT and Parvalbumin (Parv) (1:200, Chemicon). The double-labelling protocol used was similar to that described above, except that the HCl step was omitted.

### Microscopy

Basal forebrain sections of each mouse were taken at 3 different intervals, at bregma 1.18 mm, 0.98 mm and 0.74 mm, according to the mouse atlas [86] as representative samples for counting the number of MSDB cholinergic neurones. The images of ChAT-positive cells in the MSDB were captured with a digital camera (Magnafire SP, Optronics, Goleta, CA) under a 20 × objective using a BX50 microscope (Olympus, Tokyo, Japan) and counted semi-automatically (Image Pro Plus, Media Cybernetics Inc., Silver Spring, MD, USA).

For the paraffin sections, BrdU-labelled cells from one-in-five serial sections (at least 30 μm apart) throughout the rostro-caudal extent of the dentate gyrus were viewed through a 40 × objective using the BX50 microscope. Digital images were captured for the purpose of counting (Magnafire SP, Optronics). For the 4 weeks group, one-in-five sections double-labelled with either BrdU-NeuN or BrdU-GFAP were analyzed using a laser scanning confocal microscope (LSM 510, Carl Zeiss, Göttingen, Germany) under 400 × magnification using sequential illumination with 488 nm and 546 nm wavelength lasers. Colocalization was established by analyzing the overlap between the antigen expressions by orthogonal reconstruction throughout the entire z-stack and in the xy-yz direction (LSM 510, Zeiss).

### Quantification of labelled cells

The BrdU-positive cells in the granule cell layer, and their co-expression with GFAP- and NeuN- positive cells, were counted by an investigator blind to the coding. For both the 24 hr and 4 weeks group, the number of BrdU-positive cells in one side of the dentate gyrus in a section was pooled and divided by the length of the granule cell layer within that particular dentate gyrus to determine the mean number of BrdU cells per length of dentate gyrus. Sections were taken by sampling at equal intervals from the hippocampus region nearer to the septal end for more consistent BrdU labelling. This reference sample volume was 1000 μm thick. The mean number of BrdU cells per length of dentate gyrus was further divided by the thickness of the section to obtain the average number of labelled cells per traced area. The estimated number of BrdU cells in the dorsal hippocampus sample volume per brain is obtained by multiplying the average number of labelled cells per area by the mean length of the dentate gyri of the sections sampled and the reference sample volume.

### Statistical Analyses

All statistical analyses were performed using SPSS software version 14.0. Analysis of variance (ANOVA) was performed for all groups, followed by appropriate post-hoc analysis if comparisons were found to be significant. The Levene's test for Equality of Error Variances was applied to all groups to check for homogeneity of variances. Differences were considered to be statistically significant when *p *< 0.05. Data are expressed as mean ± sem.

## Authors' contributions

NFH participated in the design of the study, performed the experiments, participated in the data analysis and drafted the manuscript. SPH performed the cell counting and participated in the data collation and analysis. GSD participated in the design of the study, the data analysis and writing the manuscript. All authors read and approved the final manuscript.
